# Regulation of Proteasome Activity by (Post-)transcriptional Mechanisms

**DOI:** 10.3389/fmolb.2019.00048

**Published:** 2019-07-16

**Authors:** Suzan Kors, Karlijne Geijtenbeek, Eric Reits, Sabine Schipper-Krom

**Affiliations:** Department of Medical Biology, Amsterdam UMC, University of Amsterdam, Amsterdam, Netherlands

**Keywords:** proteasome, post translational modifications, 20S, 26S, proteasome complexes, proteasome activation/inhibition

## Abstract

Intracellular protein synthesis, folding, and degradation are tightly controlled processes to ensure proper protein homeostasis. The proteasome is responsible for the degradation of the majority of intracellular proteins, which are often targeted for degradation via polyubiquitination. However, the degradation rate of proteins is also affected by the capacity of proteasomes to recognize and degrade these substrate proteins. This capacity is regulated by a variety of proteasome modulations including (1) changes in complex composition, (2) post-translational modifications, and (3) altered transcription of proteasomal subunits and activators. Various diseases are linked to proteasome modulation and altered proteasome function. A better understanding of these modulations may offer new perspectives for therapeutic intervention. Here we present an overview of these three proteasome modulating mechanisms to give better insight into the diversity of proteasomes.

## Introduction

Protein degradation by proteasomes plays a major role in the regulation of a wide range of basic cellular processes (Rock et al., 1994). Therefore, it is not surprising that aberrations in this pathway have been linked to several diseases. Some diseases are due to the increased lifetime of disease-related proteins, whereas others are caused by accelerated protein degradation (Ciechanover and Schwartz, 2004; Hanna et al., 2019). This altered degradation capacity by the proteasome can be caused by a change in the expression of proteasome subunits or by an aberrant proteasome composition (Ciechanover and Schwartz, 2004; Dahlmann et al., 2007). Processes to enhance proteasome activity and induce expression of proteasome(-related) components have been implicated in several cancers and muscle wasting condition (Chen and Madura, 2005; Dahlmann et al., 2007; Klaude et al., 2007; Cohen et al., 2015; Zhang et al., 2015; Chen et al., 2017). In contrast, neurodegenerative disorders and cardiac dysfunction have been related to accumulation of proteins and/or decreased proteasome activity (Keller et al., 2000; Tsukamoto et al., 2006; Dahlmann et al., 2007; Dantuma and Bott, 2014; Gilda and Gomes, 2017). This emphasizes the importance of properly functioning proteasomes and the relevance for therapeutic interference. The use of proteasome inhibitors in cancer treatment is a well-known example of using the proteasome as a therapeutic target (Orlowski and Kuhn, 2008; Schlafer et al., 2017), which raises the question whether intervention in the proteasome system would also be beneficial in other diseases (Njomen and Tepe, 2019).

**Figure 1:**
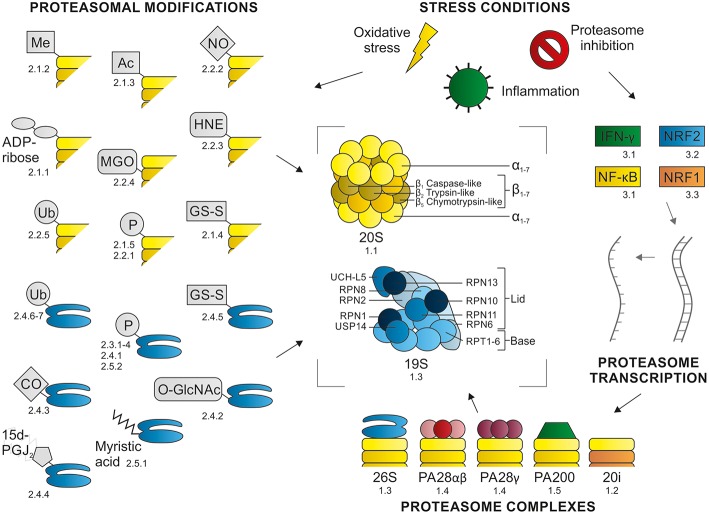
Proteasome modulations. Proteasome function can be modulated at different levels: (1) changes in proteasome complex composition, (2) post-translational modifications, and (3) alterations at transcriptional level. These modulation are induced in response to specific (stress) conditions. This figure summarizes the content of this review, with the numbers referring to the section where the modulation is described.

In order to cope with particular stress conditions, cells have their own mechanisms to inhibit and activate the proteasome. These proteasome modulations include (1) changes in the composition of proteasome complexes, (2) post-translational modifications (PTMs), or (3) alterations at the transcriptional level (Figure 1). A better understanding of these diverse endogenous modulations of the proteasome may give more insight into new possibilities for therapeutic interventions. Here we review various mechanisms used by cells to modify proteasome abundance, composition, and consequently activity.

## 1. Modulating Proteasome Activity by Changing Proteasome Composition

Proteasomes are multicatalytic complexes containing a cylindrical 20S core, which is composed of four heteroheptameric rings (Harshbarger et al., 2015). The two inner β-rings contain the six proteolytic sites where substrates are cleaved; each ring has caspase-like (C-L), trypsin-like (T-L), and chymotrypsin-like (ChT-L) activity (Figure 1). The two outer rings consist of α-subunits, which act as gatekeepers, controlling the accessibility of substrates into the catalytically active β-chamber (Kisselev et al., 2002). Proteasomes are not static complexes and the activity of the proteasome can be modulated by the binding of various proteasome activators (PAs): 19S, PA28, and PA200 (Mao et al., 2008; Savulescu and Glickman, 2011; Liu and Jacobson, 2013; Cascio, 2014). These regulators can bind symmetrically and asymmetrically to the α-rings of the 20S core, forming single or double capped proteasomes. However, the free 20S proteasome unit remains a very abundant conformation in cells (Fabre et al., 2014).

The 19S regulatory particle is the main PA, forming the 19S-20S (26S) proteasome complex (Fabre et al., 2014). This cap is essential in the ubiquitin-proteasome system (UPS); this pathway is responsible for the degradation of misfolded as well as short-lived regulatory proteins such as cell cycle regulators and transcriptional activators (Glickman and Ciechanover, 2002). Though, when mimicking starvation in cell culture via mTOR inhibition, the majority of long-lived proteins is also degraded via the UPS (Zhao et al., 2015). Folded proteins destined for degradation by the UPS are tagged by a polyubiquitin chain (Liu C. W. et al., 2006). After substrate binding to the proteasome, the 19S regulatory particle deubiquitinates, translocates and unfolds the substrate protein in an ATP-dependent manner, so that it can be degraded by the 20S core (Navon and Goldberg, 2001; Liu C. W. et al., 2006; Liu and Jacobson, 2013; Collins and Goldberg, 2017). Alternatively, the 20S proteasome can bind to PA28αβ, PA28γ, and PA200 (Rechsteiner and Hill, 2005); these PAs open the 20S core but lack deubiquitinating enzymes (DUBs) and ATPase activity. In addition, alternative forms of the 20S proteasome exist. The proteolytic active β-subunits (β1, β2, and β5) of the 20S proteasome can all or partly be replaced by so-called immunosubunits (β1i, β2i, and β5i), resulting in three 20S proteasome subpopulations: standard (or constitutive), immuno and intermediate proteasomes (Dahlmann, 2016). Finally, cell-type specific proteasome subpopulations have also been identified: thymoproteasomes (β5t) and spermatoproteasomes (α4s) which vary in catalytic activity or preference for specific PAs, respectively (Murata et al., 2007; Florea et al., 2010; Qian et al., 2013; Kniepert and Groettrup, 2014).

These various proteasome compositions can change as a consequence of various stimuli and diseases (Mishto et al., 2006; Zheng et al., 2012; Ali et al., 2013), thereby affecting their substrate specificity and the protein homeostasis in cells. In this section we give an overview of various proteasome complexes and the consequences on proteasome activity (summarized in Table 1).

**Table 1:** The different proteasome complexes with their specific properties.

**Complex**		**ATP and ubiquitin**	**Effect**	**Section**	**References**
20S	^*^	Independent	Oxidized/damaged/unfolded protein degradation	1.1	Davies, 2001; Ferrington et al., 2001; Shringarpure and Davies, 2002; Liu et al., 2003; Whittier et al., 2004; Baugh et al., 2009; Reeg et al., 2016
	-19S (26S)	Both dependent and independent	Degradation of most cellular proteins Polyubiquitinated folded protein degradation	1.3	Glickman and Ciechanover, 2002; Liu C. W. et al., 2006; Liu and Jacobson, 2013; Zhao et al., 2015; Collins and Goldberg, 2017
	-PA28αβ	Independent	Proteasome activity ↑ Changed cleavage products Short peptide degradation Oxidized protein degradation ↑	1.4	Pickering et al., 2010; Li et al., 2011; Pickering and Davies, 2012; Cascio, 2014; Lobanova et al., 2018
	-PA28γ	Independent	T-L activity ↑, ChT-L and C-L activities ↓ Changed cleavage products Cell cycle regulatory protein degradation Oxidized protein degradation ↑	1.4	Mao et al., 2008; Baugh et al., 2009; Pickering and Davies, 2012
	-PA200	Independent	Proteasome activity (mainly C-L activity) ↑; double-capped: ↓ Changed cleavage products Short peptide degradation Histone degradation Oxidized protein degradation ↓	1.5	Savulescu and Glickman, 2011; Pickering and Davies, 2012
20Si	^*^	Independent	ChT-L and T-L activities ↑, C-L activity ↓ Changed cleavage products Peptides for MHC class I antigen presentation Oxidized protein degradation	1.2	Früh and Yang, 1999; Kloetzel, 2001; Pickering et al., 2010; Seifert et al., 2010
	-19S (26i)	Both dependent and independent	Polyubiquitinated (oxidized) protein degradation Peptides for MHC class I antigen presentation	1.2	Seifert et al., 2010; Nathan et al., 2013
	-PA28αβ	Independent	Similar as PA28αβ-20S Peptides for MHC class I antigen presentation	1.4	Früh and Yang, 1999; Sijts et al., 2002; Pickering and Davies, 2012; Cascio, 2014; Raule et al., 2014
PA28αβ-20S-19S^#^		Both dependent and independent	Proteasome activity ↑ Changed cleavage products	1.4	Tanahashi et al., 2000; Cascio et al., 2002
PA200-20S-19S		-	Proteasome activity (mainly C-L activity) ↑	1.5	Blickwedehl et al., 2008

*Effect is relative to the 20S proteasome. Proteasome activity: all three proteolytic activities*;

* *uncapped*;

# * 20S and 20Si hybrids are not clearly distinguished*.

### 1.1. The 20S Proteasome

Substrate entrance into the proteolytic core of 20S proteasomes is physically blocked by the N-termini of the α-subunits (Groll et al., 2000). Binding of a PA relieves this barrier by opening the α-ring. The free 20S proteasome is therefore often described as a latent complex. Interestingly, damaged and especially oxidized proteins, which can be induced by exposure to environmental toxins, cellular stresses, diseases and aging, can be degraded by the 20S proteasome *in vitro* (Davies, 2001; Shringarpure and Davies, 2002; Whittier et al., 2004; Reeg et al., 2016). Protein oxidation results in conformational changes, and subsequently in the exposure of hydrophobic domains that were previously shielded (Ferrington et al., 2001; Lasch et al., 2001). These hydrophobic sites can bind to purified 20S proteasomes and stimulate proteasome activities by opening the barrel (Kisselev et al., 2002).

However, intracellular protein degradation by the 20S proteasome has not been clearly demonstrated (reviewed by Demasi and da Cunha, 2018). Studies suggest that the 20S proteasome can degrade oxidized proteins *in vivo* (Grune et al., 1996; Pickering et al., 2010), but direct evidence is still lacking. In response to oxidative stress the 19S regulatory particle dissociates from the 26S proteasome in yeast and mammalian cells, increasing the pool of free 20S proteasomes (Wang et al., 2010; Grune et al., 2011), which suggests a rapid mechanism to increase the capacity to degrade oxidized proteins. Though, studies show different results on whether oxidized proteins are generally ubiquitinated (Shang et al., 2001; Dudek et al., 2005; Medicherla and Goldberg, 2008) or non-ubiquitinated (Shringarpure et al., 2003; Kastle and Grune, 2011; Kastle et al., 2012), i.e., the involvement of the UPS.

Based on biochemical analysis of mammalian lysates, it was predicted that 20% of the cellular proteins is degraded by the 20S proteasome (Baugh et al., 2009). This seems a relatively high number if only damaged and oxidized proteins would be substrates for the 20S proteasome (Baugh et al., 2009). An explanation for this high number would be that the 20S proteasome also degrades native proteins. For example, p21 and α-synuclein have been linked to 20S proteasome degradation (Liu et al., 2003). Surprisingly, these proteins were even degraded *in vitro* when they lacked exposed termini. This endoproteolytic activity of the 20S proteasome was also confirmed in a study that reported cleavage in unfolded regions outside structured domains of various proteins (Baugh et al., 2009). This supports the suggestion that unfolded regions of proteins can promote gate opening and translocation into the proteolytic core. Therefore, in addition to oxidized proteins, the 20S proteasome may degrade a broad spectrum of native proteins, including tumor suppressors p21, p53, and p27 (Sheaff et al., 2000; Liu et al., 2003; Asher et al., 2005) and proteins associated with neurodegenerative diseases such as α-synuclein [Parkinson's Disease (PD)] and tau [Alzheimer's disease (AD)] (David et al., 2002; Liu et al., 2003). Though, one cannot be conclusive on this issue as most studies were performed using purified proteasomes that may degrade damaged and denatured proteins differently when compared to the UPS in living cells. Some of these proteins are indeed reported to be ubiquitinated and therefore subjected to 26S proteasome degradation. For instance, p53 is ubiquitinated and targeted for proteasomal degradation by E3 ligase MDM2 (Fang et al., 2000) and the E3 ligase CHIP was recently shown to be responsible for the ubiquitination of p21 (Biswas et al., 2017). This discrepancy may be explained by the different experimental setups, as purified proteasomes may degrade proteins independent of ubiquitination whereas intracellular degradation is largely dependent on selective protein ubiquitination followed by degradation by the 26S proteasome.

### 1.2. The Immunoproteasome

The 20S immunoproteasome (20Si) differs from the standard 20S proteasome by its proteolytic activity as the constitutive subunits β1, β2, and β5 are replaced by its immune counterparts β1i (LMP2), β2i (MECL1), and β5i (LMP7), respectively. Lymphoid tissue constitutively expresses the immunoproteasome at high levels (Sijts and Kloetzel, 2011). In non-lymphoid tissue, the immunoproteasome abundance is rather low and requires induction by cytokines, such as interferon γ (IFN-γ) (Früh and Yang, 1999; Kloetzel, 2001; Sijts and Kloetzel, 2011). The immunoproteasome has a higher ChT-L and T-L activity and lower C-L activity than the standard 20S proteasome, resulting in alternative cleavage of proteins (Gaczynska et al., 1993; Cascio et al., 2001). In general, peptides with hydrophobic or basic C-termini are generated, which are preferred by major histocompatibility complex (MHC) class I molecules that are important for the initiation of an immune response by infection (Gaczynska et al., 1993; Kloetzel, 2001).

For a long time the immunoproteasome was almost exclusively linked to peptide production for MHC class I antigen presentation. However, studies have elucidated roles for the immunoproteasome in macrophage activation and T-cell differentiation, and also in the differentiation of non-immune cells like skeletal muscle cells (Kimura et al., 2015). In addition, it has been proposed that the immunoproteasome is also involved in the preservation of general homeostasis. First, hydrogen peroxide (H_2_O_2_) treatment, which induces oxidative damage, enhanced the expression of immunoproteasomes in mouse cells (Pickering et al., 2010). Secondly, IFN-γ does not only induce immunoproteasome expression but also oxidative stress, resulting in oxidatively damaged proteins (Watanabe et al., 2003; Pickering et al., 2010; Seifert et al., 2010). Upon depletion of immunoproteasomes, formation of aggresome-like induced structures (ALIS) was accelerated in IFNγ treated cells compared to non-treated cells, indicating a role in the clearance of oxidatively damaged proteins (Seifert et al., 2010). The role of 26S immunoproteasomes in degrading oxidatively damaged proteins has however been challenged by others who did not observe improved degradation of ubiquitinated proteins by immunoproteasomes or the subsequent protective effects (Nathan et al., 2013; Lundh et al., 2017).

### 1.3. The 26S Proteasome

The 26S proteasome degrades the majority of cellular proteins and therefore plays an important role in a wide range of cellular processes, such as transcriptional regulation, the cell cycle, differentiation, DNA repair, the secretory pathway, and the biogenesis of organelles, designating the 26S proteasome as a key regulator in cellular quality control (Glickman and Ciechanover, 2002). Interestingly, it has been reported that the 26S proteasome is not very effective in degrading oxidized proteins *in vitro*, even in the presence of ATP and ubiquitin (Davies, 2001). Again, many studies were performed using purified proteasomes that may degrade damaged and denatured proteins differently when compared to the UPS in living cells.

A general overview of the most relevant 19S subunits and their function in the 26S proteasome will be discussed here. For an extensive review on the 26S proteasome's multistep degradation mechanisms we refer to Collins and Goldberg (2017) and Bard et al. (2018).

The 19S regulatory particle contains six regulatory triple-ATPase particles (RPT1–6) forming the base of the cap and 13 regulatory non-ATPase particles (RPN1–3, RPN5–13, and RPN15), which constitute the so-called lid (Figure 1) (Lander et al., 2012; Schweitzer et al., 2016). The RPN10- and RPN13-subunits are the ubiquitin-receptors, which bind ubiquitinated substrates with their ubiquitin interacting motif (UIM) or pleckstrin-like receptor for ubiquitin (PRU) domain, respectively (Elsasser et al., 2004; Husnjak et al., 2008). Recently, the RPN1 subunit was also identified as ubiquitin binding site (Shi et al., 2016). In addition to the intrinsic ubiquitin-receptors, ubiquitinated substrates can also bind to extrinsic UBL (ubiquitin-like)-UBA (ubiquitin-associated) ubiquitin-receptors, including DSK2, RAD23, and DDI1 (Elsasser and Finley, 2005). These UBL-UBA proteins interact via their UBL-domain with the proteasomal ubiquitin binding sites (Husnjak et al., 2008; Shi et al., 2016), functioning as ubiquitin shuttling proteins. However, not only the presence of ubiquitin regulates the selective and efficient degradation of proteins, the recognition of a loosely folded region also plays an important role (Peth et al., 2010).

After substrate recognition, a conformational switch of RPN11 stimulates its DUB activity, resulting in the removal and recycling of ubiquitins (Worden et al., 2017). In addition, two other DUBs, ubiquitin carboxyl-terminal hydrolase 14 (USP14) and ubiquitin carboxyl-terminal hydrolase isozyme L5 (UCH-L5) are associated with a minor pool of 26S proteasomes (de Poot et al., 2017; Kuo and Goldberg, 2017). These DUBs bind the proteasome via RPN1 and RPN10/RPN13, respectively, and trim the polyubiquitin chain into monoubiquitins and short ubiquitin chains, which consequently can either promote or prevent substrate degradation (Liu and Jacobson, 2013). Contrarily, the proteasome-associated ubiquitin-protein ligase E3C (UBE3C) extends the ubiquitin chain on substrates (Crosas et al., 2006). The exact role of ubiquitin remodeling by chain trimming and extending by these different enzymes is unclear, but it may regulate proteasome specificity (Crosas et al., 2006; Liu and Jacobson, 2013). In addition to the deubiquitinating role of USP14, it also plays a role in regulating proteasome activities. Although the 26S proteasome has a preference for polyubiquitinated proteins, the purified complex can also degrade non-ubiquitinated unfolded proteins, without ATP hydrolysis (Liu C. W. et al., 2006), but this activity is mainly inhibited by USP14 (Kim and Goldberg, 2017). When bound to ubiquitinated substrates, USP14 activates the proteasome via its UBL domain, otherwise it suppresses several activities to prevent unnecessary ATP consumption and non-specific hydrolysis (Peth et al., 2009, 2013; Kim and Goldberg, 2017, 2018). The ATP hydrolysis by the RPT-subunits in the base drives protein translocation through the α-ring gate, which forces protein unfolding (Navon and Goldberg, 2001; Kenniston et al., 2003; Snoberger et al., 2017). Gate opening is particular induced by the C-termini of RPT2 and RPT5 through binding between the 20S α-subunits (Smith et al., 2007). Though, RPT3 is also important for gate opening, since point mutations in the C-terminus inhibited gate opening of the 20S core. Other 19S-subunits are involved in stabilizing (RPN2) and structuring (RPN8) the 19S regulatory particle, or stabilizing the association between the 19S regulatory particle and the 20S core (RPN6) (Chen et al., 2016; Schweitzer et al., 2016). The role of the other subunits is not fully understood. They may support the function of other 19S-subunits (e.g., RPN3) (Chen et al., 2016; Schweitzer et al., 2016).

### 1.4. The PA28-20S Proteasome

PA28 is another regulatory particle that can associate with the 20S proteasome. The PA28 family exist of three members: PA28α, PA28β, and PA28γ. PA28α and PA28β assemble into a heteroheptamer, while PA28γ forms a homoheptamer (Mao et al., 2008; Cascio, 2014). The cap can also be formed by PA28α alone, but its affinity for and stimulation of the 20S core is lower (Huber and Groll, 2017). The localization of PA28α and PA28β, and the localization of PA28y are mutually exclusive (Wójcik et al., 1998). Whereas, PA28α and PA28β are primarily located in the cytoplasm, PA28γ is mainly present in the nucleus.

PA28αβ associates with the 20S proteasome and enhances all three proteolytic activities, stimulating its ability to degrade short peptides, rather than proteins or ubiquitinated substrates *in vitro* (Cascio, 2014). Indeed, overexpression of PA28α did not affect the turnover of some *bona fide* substrates like GATA4, AKT, and PTEN in rat cardiomyocytes or the degradation of polyubiquitinated protein/peptide substrates in retina lysates of transgenic mice (Li et al., 2011; Lobanova et al., 2018). However, PA28 overexpression does increase degradation of the UPS substrate GFPu or oxidized proteins in cells (Li et al., 2011). This is supported by *in vitro* data showing increased ability of purified proteasomes to degrade oxidized proteins in the presence of PA28αβ (Pickering et al., 2010). In addition, PA28αβ binds to the 20S proteasome immediately upon H_2_O_2_ treatment, followed by increased PA28αβ expression during oxidative stress adaptation (Pickering et al., 2010; Pickering and Davies, 2012). Therefore, PA28αβ seems to function in retaining cellular proteostasis. This is exemplified in a study on retinitis pigmentosa, where overexpression of PA28α in mice slowed retina degeneration caused by insufficient proteasome capacity to degrade misfolded mutant rhodopsin (Lobanova et al., 2018). Alternatively, the effect of PA28αβ may also be proteasome activity-independent, as chaperone-like functions have been suggested (Minami et al., 2000; Adelöf et al., 2018).

Since PA28αβ is induced by IFN-γ, the role of PA28αβ in MHC class I antigen presentation and the immune response has been studied most extensively (Tanahashi et al., 1997; Früh and Yang, 1999; Cascio, 2014). Expression of PA28αβ has been reported to selectively upregulate MHC class I antigen presentation, whereas downregulation impaired the presentation of specific antigens (Sijts et al., 2002). PA28αβ-20Si proteasomes generate higher amounts of very short products, and favors the release of several longer more hydrophilic peptides, probably by the enhanced proteolytic activities (Raule et al., 2014). While these products are not preferred by MHC class I molecules, some may be critical for an effective immune response. Mice lacking both PA28α and PA28β showed also that PA28αβ is required for the processing of certain antigens (Murata et al., 2001). However, PA28 knockout mice showed normal immune responses against an influenza virus infection, and normal disease progression during viral myocarditis, suggesting a small impact of PA28αβ in general antigen presentation (Murata et al., 2001; Respondek et al., 2017). Nevertheless, a phylogenetic analyses of proteasome subunits links the presence of PA28αβ with the IFN-γ-inducible MHC and immunoproteasome components, which would suggest an important role for PA28αβ in antigen processing (Fort et al., 2015). IFN-γ also induces the formation of PA28αβ-20S-19S hybrid proteasomes in human cells (Tanahashi et al., 2000). PA28αβ-20S-19S hybrid proteasomes generates an altered pattern of cleavage products, without altering the mean peptide length, in contrast to PA28αβ-20S proteasomes (Cascio et al., 2002; Raule et al., 2014).

PA28γ stimulates the T-L activity of the 20S proteasome, while it suppresses the other proteolytic activities (Realini et al., 1997; Mao et al., 2008). As a result, the PA28γ-20S proteasome has increased preference for cleavage after basic amino acids but enhances the degradation of short peptides only weakly. Recently, Jonik-Nowak et al. (2018) reported that in their study most PA28γ is bound to FAM192A/PIP30 in mammalian cells. This protein promotes PA28γ's interaction with the 20S core and impairs the entrance of some peptides *in vitro*, suggesting changed substrate selectivity. PA28γ-20S proteasomes can also degrade intact proteins which may occur via the cleavage of proteins in less structured domains (Baugh et al., 2009), although it remains poorly understood how these proteins can be unfolded and processed in an ATP-independent manner (Mao et al., 2008). In contrast to PA28αβ, PA28γ expression is not responsive to IFN-γ, which suggests a different role for this PA28 family member (Tanahashi et al., 1997). PA28γ is overexpressed in various type of cancers (Chai et al., 2014; Li J. et al., 2015) and several *in vivo* mice studies suggest that PA28γ is important in cell proliferation and apoptosis (Mao et al., 2008). In addition, the PA28γ-20S proteasome has been implicated in the degradation of important cell cycle regulatory proteins, including p21 and the steroid receptor SRC-3 (Li et al., 2006, 2007; Mao et al., 2008). PA28γ facilitates also the MDM2-dependent turnover of tumor suppressor p53 (Zhang and Zhang, 2008), and is involved in the regulation of chromosomal stability during mitosis (Zannini et al., 2008). Overall the PA28γ proteasome regulator is implied in cell cycle progression. PA28γ seems to have additional functions as it is recruited to sites of DNA double-strand breaks (Levy-Barda et al., 2011) and it has a role in the organization of nuclear bodies such as nuclear speckles and Cajal bodies (Cioce et al., 2006; Baldin et al., 2008). Association of PA28γ with a component of Cajal bodies is inhibited by PIP30, indicating that PIP30 can control multiple functions of the proteasome (Jonik-Nowak et al., 2018). However, the exact function of PA28γ remains unknown. There is also evidence that PA28γ enhances the ability of the 20S proteasome to degrade oxidized proteins, but in lesser extent than PA28αβ (Pickering and Davies, 2012).

### 1.5. The PA200-20S Proteasome

Like PA28γ, PA200 is a nuclear-localized proteasome regulator (Savulescu and Glickman, 2011). PA200 enhances the ability of purified 20S proteasome to degrade short peptides and unstructured proteins, especially by cleavage after acidic residues (C-L activity) (Dange et al., 2011; Savulescu and Glickman, 2011). PA200 strongly inhibited the ability of the 20S proteasome to degrade oxidized proteins *in vitro* (Pickering and Davies, 2012). The majority of PA200 regulatory particles is bound to 26S proteasomes in yeast and mammalian cells (Schmidt et al., 2005; Pickering and Davies, 2012) and in response to ionizing radiation, more PA200-hybrid proteasomes are formed, which accumulate on chromatin (Blickwedehl et al., 2008). PA200-containing proteasomes degrade acetylated core histones during DNA repair and replication stress, which is independent of ubiquitination (Qian et al., 2013; Mandemaker et al., 2018). Cells depleted from PA200 are more sensitive to DNA damage (Mandemaker et al., 2018). However, it seems that PA200 is not essential for DNA repair; in mice lacking PA200, processes that require DNA repair were not affected (Khor et al., 2006). Instead, PA200 knockout mice present with reduced fertility in male, which become complete infertile in combination with PA28γ knockout, caused by multiple defects in spermatogenesis e.g., PA200 has a role in acetylated histone degradation during spermatogenesis (Khor et al., 2006; Qian et al., 2013; Huang et al., 2016). Furthermore, PA200-capped proteasomes have been implicated in various other cell processes, such as mitochondrial fission, turnover of ribosome-related transcription factor Sfp1 and maintaining intracellular glutamine levels (Lopez et al., 2011; Blickwedehl et al., 2012; Tar et al., 2014).

## 2. Proteasome Modulation by Post-translational Modifications

Post-translational modifications (PTMs) of proteasome subunits include phosphorylation, methylation, acetylation, ubiquitination, and myristoylation. It has been shown that there is overlap between the modification sites, suggesting crosstalk in regulating proteasome function (Zong et al., 2014). To make the study of proteasome regulation by PTMs even more complex, the presence and sites of some PTMs differ between species, and there seems to be differences in PTMs between cell types and tissues. For most PTMs the specific target, effect or even relevance on proteasome functioning is unknown (Wang et al., 2007; Hirano et al., 2016). However, an increasing number of PTMs has been studied in the last decades, revealing their role in proteasome regulation. Proteasome modulation by phosphorylation has recently been reviewed by Guo et al. (2017) and VerPlank and Goldberg (2017), but an overview of all the different types of proteasomal PTMs was still missing. The following section on PTMs is subdivided in proteasome activating or inhibiting modifications of the 20S and 19S subunits (Tables 2, 3 respectively). For each PTM the class of modification is explained, followed by their consequences for proteasomal functioning and involvement in cellular processes. However, many studies show under a specific condition both altered proteasomal PTM(s) and altered proteasome activity, but a direct link between these alterations is often still lacking. In this section we will discuss PMT that are present in the 20S proteasome and PMT that are present in the 19S cap separately as to give a better overview in a broad range of modified subunits. However, post-translational modifications attributed to the 20S core could be part of larger complexes such as the 26S complex.

**Table 2:** An overview of the 20S proteasome PTMs with known target and effect.

	**Modification**		**Target**	**Cell type/ tissue**	**Enzyme(s)**	**Effect**	**Section**	**References**
20S	Activating	PolyADP-ribosylation	- (Nucleus)	Human K562 cells, mouse BV-2 and HT22 cells	PARP	ChT-L activity^*^↑, oxidized protein (e.g., histone) degradation ↑	2.1.1	Ullrich et al., 1999, 2001; Catalgol et al., 2010
		Methylation	-	Human Huh7 cells, mouse hepatocytes	-	Impaired methylation → ChT-L activity^*^↓	2.1.2	Osna et al., 2010
		Acetylation	α6, β3, β6, β7	Human and mouse myocardium	HDAC	T-L activity^*^↑	2.1.3	Wang et al., 2013
		S-glutathionylation	α5, and β-subunits	Human erythrocytes and yeast	Grx2 and Trx1/2	20S gate opening → oxidized protein degradation ↑, ChT-L activity ↓	2.1.4	Demasi et al., 2001, 2003; Silva et al., 2008, 2012; Leme et al., 2019
		Phosphorylation	α1, α2, α3, β2, β3, β7	Mouse myocardium	PKA and PP2A	Proteasome activity ↑	2.1.5	Zong et al., 2006
			α3	Human HEK293 and CA46 cells	PLK	ChT-L activity^*^↑	2.1.5	Feng et al., 2001
			α7	Human Jurkat T cells, rat α7 in monkey COS-7 cells	CKII	Stabilizing 26S proteasomes, Ecm29 binding	2.1.5	Bose et al., 2004; Schmidt et al., 2011; Wani et al., 2016
	Suppressing		α4	Human HEK293 and MCF-7 cells, mouse MEF cells	c- ABL/ARG	ChT-L activity^*^↓, ubiquitinated short-lived protein degradation ↓, α4 degradation ↓	2.2.1	Liu X. et al., 2006; Li D. et al., 2015
		S-nitrosylation	-	Rat vascular smooth muscle cells	-	Proteasome activity ↓	2.2.2	Kapadia et al., 2009
		HNE	Several α- and β-subunits	Rat heart and liver	-	Proteasome activity ↓	2.2.3	Bulteau et al., 2001; Ferrington and Kapphahn, 2004; Farout et al., 2006
		MGO	β2	Human vascular endothelial cells, mouse aorta and kidney	-	ChT-L activity ↓	2.2.4	Queisser et al., 2010
		Ubiquitination, and additional acetylation	α2	Human prostate cancer cell lines	HDAC	ALAD binding, nuclear proteasome localization	2.2.5	Schmitt et al., 2016

*In some cases only the proteasome complex is known as PTM target rather than a specific subunit. The cell types and/or tissues in which the modification is observed, as well as the involved enzymes are mentioned. (In some cases studied with purified proteasomes). Proteasome activity: all three proteolytic activities*;

*^#^neither activating nor suppressing*;

* *only measured enzymatic activity; -unknown*.

**Table 3:** An overview of the 19S related PTMs with known target and effect.

	**Modification**		**Target**	**Cell type/ tissue**	**Enzyme(s)**	**Effect**	**Section**	**References**
19S	Activating	Phosphorylation	RPT1 (Nucleus)	Human HEK293 and HeLa cells	UBLCP1	Phosphatase UBLCP1 downregulation → proteasome activity ↑, 26S proteasome assembly, ubiquitinated protein degradation ↑	2.3.1	Guo et al., 2011; Sun et al., 2017
			RPT3	Human HaCaT and MDA-MB-468 cells	DYRK2	ChT-L activity^*^↑, substrate translocation and degradation ↑	2.3.2	Guo et al., 2016; Banerjee et al., 2018
			RPT6	Human HEK293 cells, rat NRK and ST14A cells, porcine myocardium and yeast	PKA and PP1γ	ChT-L and T-L activities^*^↑, 26S proteasome assembly ↑	2.3.3	Satoh et al., 2000; Zhang et al., 2007; Lin et al., 2013; Marquez-Lona et al., 2017
				Human HEK293 cells, rat hippocampal and cortical neurons and amygdala	CaMKII	ChT-L activity^*^↑, ubiquitinated protein degradation ↑		Djakovic et al., 2009; Bingol et al., 2010; Jarome et al., 2013
			RPN6	Human HEK239 and SH-SY5Y cells, mouse C2C12 and primary hepatocytes	PKA	ATPase activity ↑, proteasome activity ↑, ubiquitinated protein degradation ↑, short-lived and aggregation-prone protein degradation ↑	2.3.4	Lokireddy et al., 2015; VerPlank et al., 2019
	Suppressing		RPT5	Human HEK293 and HeLa cells, mouse MEF cells	ASK1	RPT5 ATPase activity ↓, proteasome activity ↓, (non)ubiquitinated protein degradation ↓	2.4.1	Um et al., 2010
			RPN2	Human HeLa cells	p38 MAPK	Proteasome activity ↓, (non)ubiquitinated protein degradation ↓	2.4.1	Lee et al., 2010
		O-GlcNAcylation	RPT2	Human HUVEC cells, rat NRK cells, mouse aorta	OGT and OGA	ATPase activity ↓, ChT-L activity ↓, ubiquitinated protein degradation ↓	2.4.2	Zhang et al., 2003; Keembiyehetty et al., 2011; Liu et al., 2014
		Carbonylation	RPT3	Human SH-SY5Y cells	-	RPT3 ATPase activity ↓, ubiquitinated protein degradation ↓	2.4.3	Ishii et al., 2005
		15d-PGJ2	Several subunits	Human endothelial cells	-	ChT-L activity^*^↓, ubiquitinated protein degradation ↓	2.4.4	Marcone, 2016
		S-glutathionylation	RPN2	Human HEK293 cells and neutrophils, mouse lung	-	ChT-L and T-L activities^*^↓	2.4.5	Zmijewski et al., 2009
		Ubiquitination	RPN10	Human HEK293 cells, yeast and drosophila	RSP5 and UBP2, UBE3C, UBE3A	Substrate binding ↓, ubiquitinated protein degradation ↓, loss 26S and Dsk2 association, RPN10 degradation ↑	2.4.6	Crosas et al., 2006; Isasa et al., 2010; Piterman et al., 2014; Zuin et al., 2015; Keren-Kaplan et al., 2016
			RPN13	Human HEK293 cells	UBE3C	Substrate binding ↓, ubiquitinated protein degradation ↓	2.4.7	Besche et al., 2014
	^#^	N-myristoylation	RPT2	Yeast	-	Nuclear proteasome localization	2.5.1	Kimura et al., 2012
		Phosphorylation	RPN8	Human breast epithelial (cancer) cell lines	-	Cytoplasmic localization, loss 26S association	2.5.2	Thompson et al., 2004
			RPN3	Human HEK293 cells and mouse MEF cells	CKII (indirect)	Proteasome turnover	2.1.5	Tomita et al., 2019

*In some cases only the proteasome complex is known as PTM target rather than a specific subunit. The cell types and/or tissues in which the modification is observed, as well as the involved enzymes are mentioned. (In some cases studied with purified proteasomes). Proteasome activity: all three proteolytic activities*;

# *neither activating nor suppressing*;

* *only measured enzymatic activity; - unknown*.

### 2.1. Activating Post-translational Modifications of the 20S Proteasome

#### 2.1.1. PolyADP-Ribosylation

ADP-ribosylation is the addition of the ADP-ribose moiety of NAD^+^ to an acceptor protein (Ziegler, 2000). This covalent modification is implicated in several cellular processes, including DNA repair, apoptosis and gene regulation. Nuclear 20S proteasomes that are polyADP-ribosylated by PARP were also shown to be involved in DNA repair (Ullrich et al., 1999; Catalgol et al., 2010). H_2_O_2_-induced DNA damage activated PARP, which consequently bound DNA strand breaks and tightly interacted with proteasomes. ChT-L activity increased and the degradation of oxidatively damaged histones in the nucleus was elevated, which was dependent on the activation of the nuclear 20S proteasome by polyADP-ribosylation (Ullrich et al., 1999). In this way, proteasomes recognize and degrade the oxidized histones, which will otherwise cross-link with the DNA, making DNA repair impossible. Since antitumor chemotherapy generally causes oxidative stress in the nucleus, and subsequently DNA damage, polyADP-ribosylation of the nuclear 20S proteasome might be an adaptive response, and may be partly responsible for the development of long-term resistance to many of these drugs (Ozben, 2007). Therefore, PARP-inhibitors might improve antitumor chemotherapeutic treatment.

In addition, proteasome modification via ADP-ribosylation was also shown to be involved in neuroinflammation (Ullrich et al., 2001). Activated microglial cells release free radicals which can lead to neuronal cell death, which may have a role in neurodegenerative diseases (Liu and Hong, 2003). Microglial cells are more resistant toward free radicals. TNF-α induced activation of mouse microglial cells resulted in increased proteasomal degradation of an oxidatively damaged model substrate in lysates (Ullrich et al., 2001). This enhanced nuclear proteasome activity in activated microglial cells was dependent on active PARP, thereby protecting activated microglia from protein oxidation and cell death. Although the enhanced activity was attributed to the interaction between active PARP and the nuclear proteasome, polyADP-ribosylation of the nuclear 20S proteasome by PARP seems likely. In conclusion, the nuclear 20S proteasome can be polyADP-ribosylated by PARP, resulting in increased proteasome activity, which is probably reflected in the enhanced ability to degrade oxidized proteins, including histones.

#### 2.1.2. Methylation

S-adenosylmethionine (SAM) is the principal methyl (-CH_3_) donor for methylation in many biological processes, and therefore, indicates the methylation potential of a cell (Chiang et al., 1996). It has been shown that ethanol exposure leads to a decrease in the methylation potential, and that this inhibits the ChT-L activity of the proteasome in mouse hepatocytes (Osna et al., 2010). Exposure of human hepatoma cells to a methylation inhibitor had a similar effect and incubation of purified 20S proteasomes at relatively low SAM levels reduced lysine methylation of the complex (Osna et al., 2010). This suggests that proteasome activity is directly regulated by the methylation potential via proteasomal subunits or via co-purified proteins with a SAM-dependent methyltransferase-like activity.

Since the methylation potential can be influenced by ethanol, the impaired proteasome activity due to a changed methylation state may be involved in the development and/or progression of diseases associated with alcohol consumption. Indeed a study by Bardag-Gorce et al. (2006) found an ethanol induced decrease in proteasome activity, leading to the formation of protein aggregates (Mallory bodies) in patients with alcoholic liver disease (ALD). Therefore, methyl group donors, such as SAM, might be potential as treatment to reverse the proteasome inhibition by correcting the methylation potential in the cells of ALD patients (Osna et al., 2010). In addition, alcohol abuse accelerates the progression of hepatitis C (HCV) infection, and increases the risk of death (Safdar and Schiff, 2004). Reduced proteasome methylation induced by alcohol consumption, is suggested to have a role in the accelerated pathogenesis since the decreased proteasome activity can dysregulate antigen presentation, and therefore the recognition of HCV infected cells by the immune system (Osna et al., 2012). This is further supported by the observation that immunoproteasomes seem to be more inhibited at low SAM levels than 20S proteasomes (Osna et al., 2010). Furthermore, the methylation potential was lower in ethanol-fed HCV^+^ mice than in ethanol-fed HCV^−^ mice (Osna et al., 2012). This emphasizes the accumulating negative effect of ethanol on liver with inflammation. In short, an impaired cellular methylation potential suppresses proteasome activity, which is associated with pathogenesis.

#### 2.1.3. Acetylation

Acetylation is the substitution of an acetyl group (–CH_3_CO) for an active hydrogen atom, and is an important modification of proteins in diverse cellular processes (Choudhary et al., 2009). It plays a central role in the control of gene expression, regulated by histone acetyltransferases (HATs) and histone deacetylases (HDACs), which add and remove acetyl groups from lysine residues, respectively (Verdone et al., 2005).

These enzymes can also affect the proteasome; HDAC inhibitors enhanced the acetylation of the 20S proteasome, which correlated with an increase in the T-L activity of the proteasome in mouse and human myocardium (Wang et al., 2013). Examination of the acetylome of purified proteasomes of mouse myocardium treated with HDAC inhibitors *in vivo* revealed the inducible acetylation of α6 (Lys-30 and Lys-115), β3 (Lys-77), β6 (Lys-203), and β7 (Lys-201). The regions of these lysine residues are conserved in human (Wang et al., 2013).

Cardiac ischemia/reperfusion (I/R) injury is associated with suppressed proteasome activity, in which HNE modifications may have a role (section 2.2.3) (Bulteau et al., 2001). HDAC inhibition restored the proteasome activity in acutely I/R injured mice and end-stage ischemic failing human myocardium (Wang et al., 2013). Therefore, HDAC inhibitors might be potential drugs for regulating the proteasomal function in injured hearts. In summary, HDAC inhibitors enhance the proteolytic activity of the proteasome, likely by increased acetylation of 20S-subunits, although direct evidence for increased acetylationis lacking.

#### 2.1.4. S-glutathionylation

S-glutathionylation is the reversible formation of disulfides (-S-S-) between the thiol group (-SH) of glutathione (GSSG or GSH) and cysteine residues, which can be activated by oxidants (Hill and Bhatnagar, 2012). Upon H_2_O_2_ treatment, yeast 20S proteasomes were S-glutathionylated both *in vitro* and *in vivo* (Demasi et al., 2003). This resulted also in decreased proteolytic proteasome function, especially the ChT-L activity. The ChT-L activity was also affected by addition of GSH (mM), but not by GSSG *in vitro* (Demasi et al., 2003). The activity of mammalian proteasomes was modulated by both glutathione redox forms; low concentrations (μM) of GSH or GSSG increased, and high concentrations (mM) of GSH or GSSG decreased the ChT-L activity (Demasi et al., 2001).

In contrast to reduced ChT-L activity, the degradation rate of oxidized and partially unstructured proteins was higher by the S-glutathionylated form of the purified yeast 20S proteasome than the reduced form (Silva et al., 2012). Examination of the S-glutathionylation state of the yeast proteasome revealed modified cysteine residues of the α5-subunit, of which Cys-76 is highly conserved from yeast to human. When this residue is S-glutathionylated, the 20S proteasome is in its maximal open gate conformation, increasing the accessibility for oxidized proteins (Silva et al., 2012; Demasi et al., 2014; Leme et al., 2019). Although the S-glutathionylated cysteine residues in the β-subunits could not be identified, S-glutathionylation of the proteasomal catalytic site promoted an allosteric modification, leading to changes in the length of the 20S proteasome, thereby probably inhibiting the ChT-L acticity (Silva et al., 2012). This last mechanism may also support the function of irreversible proteasome inhibitors, which increase the S-glutathionylation of purified human 20S proteasomes (Demasi et al., 2001). It is suggested that the binding of these inhibitors leads to a conformational change, opening the 20S proteasome, and subsequently allowing S-glutathionylation. This is in agreement with increased GSH incorporation in the proteasome upon heat-denaturation and treatment with detergents, which both trigger gate opening (Demasi et al., 2001). S-glutathionylation of the 20S proteasome is a reversible modification (Silva et al., 2008). The oxidoreductases glutaredoxin 2 and thioredoxins are able to enter the core particle, remove the S-glutathionylation and allow recovery of the proteolytic activity. In summary, S-glutathionylation of the 20S proteasome triggers gate opening, which likely increases the degradation of oxidized proteins, but reduces the ChT-L activity.

#### 2.1.5. Phosphorylation

Protein phosphorylation is the addition of a phosphate group (P${\text{O}}_{4}^{3-}$) to an amino acid residue, and is important in almost every cellular process (Cohen, 2002). Phosphorylation and its counterpart dephosphorylation are catalyzed by kinases and phosphatases, respectively, regulating protein function. The phosphorylation of several 20S-subunits can be regulated by cAMP-dependent protein kinase (PKA) and protein phosphatase 2A (PP2A). Active PKA enhanced the serine phosphorylation of the α1-, α2-, α3-, β2-, β3-, and β7-subunits, and the threonine phosphorylation of the α3-, β3-, and β7-subunits of purified mouse cardiac 20S proteasomes (Zong et al., 2006). These modifications elevated all three proteolytic activities of the 20S proteasome. The same study also showed that PP2A reduced serine phosphorylation on α1 and β7, and threonine phosphorylation on α1, which was linked to suppressed proteasome activity. It is not clear which role the different subunits have in the altered proteasome activity, but the study suggests that in general proteasome phosphorylation and dephosphorylation are associated with increased and decreased proteasome activity, respectively. In addition, Hirano et al. (2016) identified multiple phosphorylation sites on almost all subunits in yeast, though the question remained whether these sites are all functional.

In addition to PKA, α3-subunits can be phosphorylated by Polo-like kinase (PLK) (Feng et al., 2001). PLK interacted with 20S (and 26S) proteasomes, and subsequently, phosphorylated α3 in human cells, which resulted in higher ChT-L activity of the proteasome.

Phosphorylation seems a constitutive modification of the α7-subunit (Gersch et al., 2015). Casein kinase II (CKII) phosphorylates α7 at Ser-243 and Ser-250 (Bose et al., 2004). After IFN-γ treatment of monkey kidney-fibroblast cells, the α7 phosphorylation decreased, resulting in destabilization of the 26S proteasome (Bose et al., 2004). Decreased 26S proteasome levels were accompanied with increased levels of PA28-proteasomes. Thus, the α7 phosphorylation state may be involved in stabilizing the association of the 19S cap with the 20S core, and therefore, the regulation of proteasome complexes. The destabilizing effect of α7 dephosphorylation on 26S proteasomes may also have a role in binding to Ecm29, a proteasome quality control factor (Wani et al., 2016), and during apoptosis (Schmidt et al., 2011). Another target of CKII is RPN3, however this modification seems to be involved in the turnover of proteasomes (Tomita et al., 2019). Finally, the kinase Aurora B is now also identified as an enhancer of proteasome activity in cell cycle regulation. Although evidence for proteasome phosphorylation was not shown, a direct effect of Aurora B on the (26S) proteasome was demonstrated by interaction studies and *in vitro* activation (Fan et al., 2019).

### 2.2. Suppressing Post-translational Modifications of the 20S Proteasome

#### 2.2.1. Phosphorylation

Phosphorylation of the α4-subunit by tyrosine kinases c-ABL and ARG has diverse effects (Liu X. et al., 2006; Li D. et al., 2015). First, phosphorylation at Tyr-153 (and maybe also at Tyr-106) led to the inhibition of the ChT-L activity of the 20S and 26S proteasome, and decreased degradation of ubiquitinated short-lived proteins by the 26S proteasome in human and mouse cells (Liu X. et al., 2006; Li D. et al., 2015). Activation of c-ABL by H_2_O_2_ or γ-irradiation increased its interaction with α4, and inhibited proteasome function (Liu X. et al., 2006). Expression of a phospho-dead α4 mutant at Tyr-153 in human cells resulted in downregulation of several cell cycle regulatory proteins, and G1/S cell cycle arrest, highlighting the role of proteasome tyrosine phosphorylation by c-ABL/ARG in cell cycle control.

Secondly, phosphorylation of α4 at Tyr-106 by c-ABL/ARG protected the 20S subunit from degradation due to suppressed polyubiquitination (Li D. et al., 2015). In addition, c-ABL/ARG upregulated α4, thereby increasing cellular proteasome abundance, under normal and oxidative stress conditions. This is consistent with the observation that cells expressing a BCR-ABL construct, a model for myeloid leukemia cells, had higher proteasome levels (Magill et al., 2004). However, this seems contradictory with the described decreased proteasome activity (Liu X. et al., 2006; Li D. et al., 2015). The authors explained the dual role of c-ABL/ARG on α4 via phosphorylation by the fact that the regulation is time-course dependent (Li D. et al., 2015). During oxidative stress, activated c-ABL/ARG initially inhibits the proteasome, preventing the degradation of short-lived regulatory cell cycle proteins, such as p53, and thereby inducing cell cycle arrest to prevent mitosis of oxidatively damaged cells. Although, this seems in disagreement with the observed G1/S cell cycle arrest in the absence of Tyr-153 phosphorylation (Liu X. et al., 2006). Meanwhile, the proteasome abundance is gradually increased via c-ABL/ARG to degrade oxidized proteins (Li D. et al., 2015). Thus, phosphorylation of the α4-subunit has various effects; it compromises proteasome activity and/or prevents ubiquitin-proteasome degradation of this subunit.

#### 2.2.2. S-nitrosylation

S-nitrosylation is the transfer of a nitric oxide (NO) moiety on a free thiol group (-SH) of a protein to form nitrosothiol (-SNO) (Broillet, 1999). It was shown that recombinant 20S core particles can be S-nitrosylated at 10 cysteine residues (Kapadia et al., 2009). These modifications provide a mechanism where NO suppresses all three proteolytic activities of the 26S proteasome in rat vascular smooth muscle cells (VSMCs) (Kapadia et al., 2009). However, identification of the specific modified cysteine residues and mutational studies should provide evidence whether S-nitrosylation of the proteasome is indeed causing the reduction in proteasome activity upon NO exposure *in vivo*. NO induces the synthesis of cGMP and the resultant activation of GSK, which has been shown to enhance proteasome activity (Ranek et al., 2013), but inhibition of cGMP/cAMP synthesis or PKG/PKA did not affect the NO-mediated inhibition (Kapadia et al., 2009). In addition to the affected proteasome activity, the expression of the α5-, α6-, β1-, and β1i-subunits increased following NO exposure. This seems contradictory, because the C-L activity regulated by the β1-subunit was the most inhibited proteolytic activity in VSMCs exposed to NO. This increased expression might be an indirect response to the inhibition (a common autoregulatory process; see section 3.3), to limit the effect of NO by synthesis of additional proteasomes, and hence overcoming the suppression of the C-L activity. Since it was shown that NO inhibits the proteasome, and the proteasome regulates the cell cycle through protein degradation, it is likely that NO produced by endothelial cells suppresses the proliferation of VSMCs through S-nitrosylation of the 20S proteasome (Kapadia et al., 2009). Overall, NO can reversibly inhibit the 26S proteasome possibly by S-nitrosylation of the 20S core.

#### 2.2.3. 4-hydroxy-2-nonenal Modification

4-hydroxy-2-nonenal (HNE), an α,β-unsaturated aldehyde (-CHO), is generated during lipid peroxidation by free radicals in response to oxidative stress (Esterbauer et al., 1991). HNE can react with cysteine, histidine and lysine residues to form a mixture of adduct types (which is a form of carbonylation; see section 2.4.3). Multiple 20S proteasome subunits have been identified that can be modified by HNE. Purified rat cardiac 20S proteasomes appeared to be modified after HNE treatment on the α1-, α2-, α4-, α5-, α6-, and β6-subunits (Farout et al., 2006). Another study found that three of these subunits (α1, α2 and α4) were HNE-modified in rat myocardium after I/R injury (Bulteau et al., 2001). In both studies, T-L activity of the purified cardiac 20S proteasomes was suppressed (Bulteau et al., 2001; Farout et al., 2006). Loss of ChT-L and C-L activities were observed after incubation at higher HNE concentrations and in cytosolic extracts of I/R injured myocardium (Bulteau et al., 2001; Ferrington and Kapphahn, 2004; Farout et al., 2006). Thus, it seems likely that in I/R injured myocardium the proteasome activity is reduced due to HNE modification.

The modification sites and the effects of HNE on the proteasome in heart differ from that in liver. Purified rat liver 20S proteasomes appeared to be modified after incubation with HNE on the α2-, α3, α4-, α5-, and β4-subunits, and at higher HNE concentrations also on the β3- and β1i-subunits (Farout et al., 2006). Another study observed a modification of α6, and suggested that α2 and α4 were already HNE-modified *in vivo* (Ferrington and Kapphahn, 2004). The ChT-L activity was reduced at low HNE concentrations, and inactivated rapidly, while for inhibition of the other proteolytic activities a higher concentration or prolonged exposure was required (Ferrington and Kapphahn, 2004; Farout et al., 2006). Thus, although the HNE-modification sites seem to be tissue specific and condition dependent, it generally results in downregulation of proteasome activity.

#### 2.2.4. Methylglyoxal Modification

The reactive dicarbonyl methylglyoxal (MGO; CH_3_C(O)CHO) is a side-product of several metabolic pathways, with glycolysis as most important source (Allaman et al., 2015). MGO is one of the most potent glycating agents present in cells, and reacts with molecules, including lysine and arginine residues of proteins to form advanced glycation end products (AGEs), such as carboxyethyllysine and methylimidazolone, respectively (which is a form of carbonylation; see section 2.4.3). Normally, MGO is detoxified, but MGO levels are increased under intracellular hyperglycemia, a condition observed with diabetes mellitus (DM) (Queisser et al., 2010). Incubation of human vascular endothelial cells with high glucose or MGO reduced the proteasomal ChT-L activity, but the other proteolytic proteasome activities were not affected (Queisser et al., 2010). Downregulation of proteasome activity was also observed in kidneys of diabetic mice, and mice that exhibit high MGO levels, confirming that MGO alone can cause proteasome inhibition. In both mouse models MGO modification (methylimidazolone) of β2 was detected. However, the alteration in proteasome activity is tissue specific; in kidney of diabetic mice all three proteasome activities were reduced, while cardiac proteasome activity was not changed (Queisser et al., 2010). In addition, another important observation is that high glucose and MGO levels both reduced 19S protein content in cells. Overall, MGO can modify the proteasome, resulting in decreased activity.

#### 2.2.5. Ubiquitination and Additional Acetylation of the α2-Subunit

Ubiquitination is the attachment of ubiquitin, a small 76-residue polypeptide, to lysine residues of protein substrates (Pickart, 2001). Although polyubiquitination is involved in the selective degradation of proteins by the proteasome, this modification can also affect proteins in other ways, such as altering activity, protein interactions, and cellular localization. The 20S proteasome can also be regulated by ubiquitination. δ-aminolevulinic acid dehydratase (ALAD) interacts with the 20S proteasome via ubiquitinated α2 in human cells and this interaction is enhanced after HDAC inhibition (Schmitt et al., 2016). Contradicting effects of ALAD on proteasome activity are reported. It was found that ALAD enhanced the ChT-L and T-L activities of 20S proteasomes purified from rat liver (Bardag-Gorce and French, 2011), whereas other studies observed that ALAD inhibited the degradation of a proteasome substrate and reduced the ChT-L in human cells (Guo et al., 1994; Schmitt et al., 2016). Since ALAD has been shown to be identical to proteasome inhibitor CF-2, it seems likely that the enzyme suppresses proteasome activity (Guo et al., 1994). ALAD might block the entrance of substrates into the 20S core.

### 2.3. Activating Post-translational Modifications of the 19S-Subunits

#### 2.3.1. Phosphorylation of RPT1

The 26S proteasome is, like the 20S proteasome, regulated by phosphorylation. The phosphatase UBLCP1 has been shown to interact with the 19S regulatory particle via RPN1, preferentially of nuclear proteasomes (Guo et al., 2011; Sun et al., 2017). A dephosphorylation screen showed that RPT1 was the only 19S subunit that was dephosphorylated (Sun et al., 2017). As a consequence the ATPase activity of RPT1 was impaired. This resulted in the inhibition of the ChT-L activity and negatively regulated the assembly of the 26S proteasome *in vitro* and *in vivo* (Guo et al., 2011; Sun et al., 2017). Previously, it was reported that RPT1 with ATP binding mutations was unable to be incorporated into the 26S proteasome, showing that the ATPase activity is essential for 26S complex assambly (Liu C. W. et al., 2006; Kim et al., 2013). Furthermore, downregulation of UBLCP1 enhanced all three proteolytic activities and (polyubiquitinated) protein degradation in the nucleus (Guo et al., 2011). Therefore, UBLCP1 regulates the assembly of 26S proteasomes via dephosphorylation of RPT1.

#### 2.3.2. Phosphorylation of RPT3

The RPT3-subunit is phosphorylated in a cell cycle-dependent manner: phosphorylation of Thr-25 by DYRK2 was low during G1 phase, became upregulated when cells transit form G1 to S phase, and remained thereafter constant in human cells (Guo et al., 2016). Thr-25 phosphorylation increased substrate-stimulated ATP-hydrolysis, without changing basal ATPase activity, indicating that the modification promotes substrate translocation and degradation (Guo et al., 2016). Overexpression of DYRK2 downregulated cell cycle inhibitors in human cells. Therefore, it is likely that increased RPT3 phosphorylation in cells entering the S phase results in the degradation of cell cycle inhibitors, promoting cell cycle progression. In addition, blocking RPT3 Thr-25 phosphorylation or knocking down DYRK2 resulted in slower proliferation of human cells, while overexpression of DYRK2, as seen in several cancer types (Santarius et al., 2010), showed opposite results (Guo et al., 2016). Interestingly, curcumin was recently identified to specifically inhibit DYRK2, diminishing RPT3 Thr-25 phosphorylation in human cells (Banerjee et al., 2018). The curcumin treatment inhibited all three proteolytic activities of the proteasome, impairing cell proliferation with induction of apoptosis, and resulted in reduced tumor growth in mice. In summary, RPT3 phosphorylation leads to increased substrate translocation into the proteasome and subsequent degradation, playing an important role in cell proliferation.

#### 2.3.3. Phosphorylation of RPT6

Both PKA and Calcium-calmodulin-dependent protein kinase II (CaMKII) have been identified to phosphorylate the RPT6-subunit at Ser-120 (Zhang et al., 2007; Djakovic et al., 2009). It has been suggested that RPT6 can also be phosphorylated by PKG; Ranek et al. reported an acidic shift of RPT6 and β5 upon PKG activation in cardiac cells, although the phosphorylated residues were not identified (Ranek et al., 2013, 2014). In human cells, endogenous RPT6 was already phosphorylated at basal state, which increased after PKA activation (Zhang et al., 2007). Activated PKA stimulated the ChT-L and T-L activity of proteasomes in rat cells, and of purified 26S proteasomes (Zhang et al., 2007). How the modification affects proteasome function is unknown, but it may lead to a conformational change, enhancing the diffusion of small peptide substrates into the 20S core. The RPT6 phosphorylation may also initiate 26S assembly, by stimulating the association of the 19S particle with the 20S proteasome as shown in porcine cells (Satoh et al., 2000). Protein phosphatase 1γ (PP1γ) could reverse the RPT6 phosphorylation, and the effect of PKA on proteasome activity (Zhang et al., 2007). However, other phosphatases may also remove the modification.

In a mouse model for Huntington's Disease (HD), activation of PKA and a phospho-mimetic Ser-120 mutant both reduced mHTT aggregates, indicating increased proteasome activity (Lin et al., 2013). Furthermore, in a yeast HD model aggregates were larger in a phospho-dead Rpt6Ser-119 (Ser-120 in mammals) strain, which showed decreased proteasome activity (Marquez-Lona et al., 2017). This suggests that phosphorylation at Ser-119/120 has a role in counteracting proteotoxic stress including protein aggregation. Importantly, other studies could not identify phosphorylation of RPT6 by PKA *in vitro* and *in vivo* (Lokireddy et al., 2015; VerPlank et al., 2019). Decreased aggregation in the HD mouse model after PKA activation could therefore be a consequence of RPN6 phosphorylation (as discussed in section 2.3.4).

It was also reported that CaMKII phosphorylates RPT6 at Ser-120, which resulted in increased proteasome activity in rat neurons and human cells (Djakovic et al., 2009). Learning-induced enhancement of proteasome activity was associated with elevated phosphorylation of RPT6 Ser-120 by CaMKII, but not PKA, in the amygdala of rats (Jarome et al., 2013). The possible role of this modification in the formation of long-term memories has been shown in other studies; after neuronal activation, autophosphorylated CaMKII functioned as a scaffold to recruit proteasomes to dendritic spines, and increased their (ChT-L) activity by phosphorylating RPT6, leading to the degradation of polyubiquitinated proteins (Bingol et al., 2010). The enhanced RPT6 phosphorylation was sufficient to change synaptic strength and induce dendritic spine outgrowth (Djakovic et al., 2012; Hamilton et al., 2012). These findings suggest that CaMKII regulates proteasome activity in neurons. In conclusion, phosphorylation of RPT6 stimulates proteasome activity, and although the same residue is phosphorylated, the responsible kinase seems to be condition/cell type dependent.

#### 2.3.4. Phosphorylation of RPN6

PKA is also responsible for the phosphorylation of RPN6 at Ser-14 (Lokireddy et al., 2015; VerPlank and Goldberg, 2017). Activated PKA promoted degradation of short-lived proteins, such as misfolded and regulatory proteins, and aggregation-prone proteins in soluble and insoluble state, associated with amyotrophic lateral sclerosis (ALS) and AD, in human cells (Lokireddy et al., 2015; VerPlank et al., 2019).

The increased degradation of ubiquitinated proteins is probably the result of the observed enhancement in ATPase activity. The stimulatory effect of PKA via RPN6 was confirmed with a phospho-mimetic RPN6 mutant (Lokireddy et al., 2015). In addition, all three proteolytic activities of purified 26S proteasomes were enhanced from cells treated with pharmacological agents that raise cAMP to activate PKA (Lokireddy et al., 2015). Raising cAMP levels also slightly increased the amount of double-capped 26S proteasomes, which suggests that RPN6 phosphorylation increases the association and stabilization of these complexes (Pathare et al., 2012; VerPlank et al., 2019).

All these findings show that activation of PKA may be useful in the treatment of neurodegenerative diseases to stimulate and increase the degradation of aggregation-prone proteins. Studies have already shown that raising cAMP levels reduced the aggregation of both mutant tau in a mouse model of tauopathy (associated with AD) (Myeku et al., 2016), and the aggregation of mutant huntingtin in an HD mouse model (as discussed in section 2.3.3) (Lin et al., 2013). Importantly, VerPlank et al. (2019) demonstrated that RPN6 phosphorylation and the consequent increased proteolysis is also initiated in response to various hormones and physiological conditions that raise cAMP, showing that cells can rapidly adapt to changing conditions when necessary.

### 2.4. Suppressing Post-translational Modifications of the 19S-Subunits

#### 2.4.1. Phosphorylation

In contrast to the often observed increases in proteasome activity upon phosphorylation, phosphorylation of the RPT5-subunit reduces proteasome activity. Apoptosis-regulating kinase ASK1, a member of the MAP3K family, interacted with the 19S particle and phosphorylated RPT5 in human cells, thereby inhibiting the ATPase activity (Um et al., 2010). All three proteolytic activities of the proteasome were reduced in cells overexpressing ASK1. In addition, the degradation of poly- and nonubiquitinated substrates was slower. ASK1 negatively regulates the 26S proteasome under stress condition, since the enzyme was activated by H_2_O_2_ and an apoptosis-inducer, causing decreased 26S proteasomal activity in mouse cells (Um et al., 2010). Therefore, phosphorylation of RPT5 inhibits proteasome activity and seemingly plays a role in apoptosis.

The RPN2-subunit is phosphorylated by the kinase p38 MAPK (Lee et al., 2010). In human cells, p38 MAPK was activated by hyperosmotic stress, resulting in the phosphorylation of RPN2 at Thr-273, and stabilization of poly- and nonubiquitinated substrates (Lee et al., 2010). Purified 26S proteasomes from cells expressing activated p38 MAPK, had a reduction of all three proteolytic activities. Since a phospho-dead RPN2 mutant at Thr-273 antagonizes the inhibitory effect of p38 MAPK, it is likely that the modification on RPN2 plays an important role in the proteasome inhibition. Though, it was also reported that inhibition of the p38 MAPK pathway (by specific MAPK or MAP2K inhibitors) did induce increased proteasome activity but no difference in the phosphorylation state of the proteasome, including RPN2 Thr-273 (Leestemaker et al., 2017). The link between RPN2 and altered proteasome activities does not seem immediately clear, because RPN2 seems to be a scaffold for other proteasome subunits (Schweitzer et al., 2016). However, it is indicated that RPN2 interacts with 19S ATPases (Schweitzer et al., 2016), which regulate gate opening by their C-termini (Smith et al., 2007). The phosphorylation of RPN2 may cause a conformational change, affecting the accessibility of substrates to the 20S core via the ATPases. Therefore, phosphorylation of RPN2 negatively regulates proteasome activity.

#### 2.4.2. O-GlcNAcylation

Protein O-GlcNAcylation is a form of glycosylation involving the addition of O-linked β-N-acetylglucosamine (O-GlcNAc) at serine and threonine residues (Love and Hanover, 2005). O-GlcNAc is derived from the hexosamine biosynthetic pathway, a nutrient-sensing pathway. The addition of O-GlcNAc is catalyzed by O-GlcNAc transferase (OGT). Exposure of purified mammalian 26S proteasomes to this transferase resulted in reduced ATPase and ChT-L activity of the proteasome, but not the T-L activities (Zhang et al., 2003). Human and rat cells treated with glucosamine (GlcN), which activates the hexosamine pathway, showed enhanced RPT2 O-GlcNAcylation and decreased proteasome ChT-L activity (Zhang et al., 2003; Wang et al., 2009; Liu et al., 2014). The GlcN-suppressed proteasome activity could be restored by O-GlcNAcase (OGA), which removes the O-GlcNAc (Zhang et al., 2003; Liu et al., 2014).

Downregulation of OGA resulted in accumulation of polyubiquitinated proteins and reduced the ChT-L activity of proteasomes in human cells (Keembiyehetty et al., 2011). Consistent with this, overexpression of OGA resulted in opposite findings (Liu et al., 2014). In both conditions, the T-L activity was not affected (Keembiyehetty et al., 2011; Liu et al., 2014). This effect on specific proteasome activities is in agreement with the mentioned effect of OGT (Zhang et al., 2003). The authors assign this selectivity to peptide hydrophobicity; since T-L and C-L substrates are more hydrophilic, they may not require the opening function of RPT2 to enter the 20S core.

Since O-GlcNAc is seen as a nutritional sensor, it may serve as a mechanism to control the availability of amino acids and regulatory proteins to metabolic changes, such as nutrient overload and starvation, by affecting proteasome activity via RPT2 modification (Zhang et al., 2003; Zachara and Hart, 2004). In addition, O-GlcNAcylation of the proteasome might play a role in lipid droplet metabolism, since a lipid droplet-associated OGA isoform and the proteasome regulated each other in a negative feedback loop (Keembiyehetty et al., 2011). Unfortunately, RPT2 O-GlcNAcylation was not analyzed in this study. In addition to the role of O-GlcNAcylation in metabolism, it has been shown that this modification on RPT2 can also be induced by NO in human vascular endothelial cells, which was confirmed in aortic tissue of mice (Liu et al., 2014). The subsequent reduced proteasome activity, similar as with OGT, may maintain, together with other NO-mediated PTMs such as the above described S-nitrosylation (section 2.2.2), the basal low proteasome activity in vascular endothelial cells (Kapadia et al., 2009; Liu et al., 2014). Together, several studies confirm that RPT2 can be modified by O-GlcNAcylation, which decreases proteasome activity.

#### 2.4.3. Carbonylation

Carbonylation is an irreversible oxidative modification, which implies the introduction of a carbonyl group (-CO) into a protein (Dalle-Donne et al., 2006; Madian and Regnier, 2010). Protein carbonyl groups can be generated indirectly by forming adducts with lipid peroxidation products (e.g., aldehyde HNE; section 2.2.3) or reactive carbonyl derivatives produced by the reaction of reducing carbohydrates (e.g., ketoaldehyde MGO; section 2.2.4), or directly by oxidative cleavage of the protein backbone or amino acid side chain with free radicals. Direct carbonylation can be induced by 15-deoxy-Δ^12, 14^-prostaglandin J_2_ (15d-PGJ_2_; see section 2.4.4), and caused RPT3 carbonylation in human neuroblastoma cells (Ishii et al., 2005). This modification impaired RPT3 ATPase activity, and decreased degradation of ubiquitinated proteins in cell lysates, accompanied with enhanced accumulation of these proteins. In conclusion, carbonylation of proteasomes seems to have a negative effect on 26S proteasome activity.

#### 2.4.4. 15-Deoxy-Δ12,14-prostaglandin J_2_ Modification

15d-PGJ_2_ is an active lipid compound derived from the prostaglandin PGD2 (Surh et al., 2011). The α,β-unsaturated carbonyl group located in the cyclopentenone ring can form a covalent bond with the thiol group of cysteine residues. Treatment of human aortic endothelial cells with 15d-PGJ_2_ resulted in 15d-PGJ_2_-modified 19S-subunits, e.g., RPN1, RPN2, RPN3, and RPN6, while 20S-subunits were unmodified (Marcone, 2016). Further examination showed that the ChT-L activity of the proteasome was inhibited and ubiquitin-conjugated proteins were accumulated. 15d-PGJ_2_ has anti-inflammatory actions, for instance by inhibiting nuclear factor-κB (NF-κB) activation (Surh et al., 2011). A possible mechanism for 15d-PGJ_2_ to suppress the NF-κB pathway is by modifying and inhibiting the proteasome, which regulates the processing of NF-κB inhibitors (Marcone, 2016). 15d-PGJ_2_ treatment reduced the adhesion and migration of monocytes toward TNF-α-exposed endothelial cells, a key process in vascular inflammation, and similar effects were observed upon proteasome inhibition. This suggest that 15d-PGJ_2_ regulates inflammatory processes by modifying proteasomal 19S-subunits.

#### 2.4.5. S-glutathionylation

The RPN2-subunit can be S-glutathionylated, which can be induced by incubation of purified human 26S proteasomes with both GSH and H_2_O_2_, causing decreased ChT-L and T-L activities (Zmijewski et al., 2009). The S-glutathionylation of RPN2 was also observed after exposure of human cells to H_2_O_2_ as well as in lung extracts of mice with enhanced intracellular H_2_O_2_ levels. RPN2 may directly affect the 20S activity, since incubation of purified human 20S proteasomes with non-oxidized (cysteine residues of) RPN2 enhanced its activity, which was not observed with oxidized RPN2 (Zmijewski et al., 2009). Although it was demonstrated that RPN1 was also S-glutathionylated in the above described conditions, there was no significant difference in 20S proteasome activity between oxidized and non-oxidized RPN1. Besides the direct effect on 20S activity, it is also possible that S-glutathionylation of RPN2 changes its interaction with other 19S subunits, resulting in reduced substrate supply, entry, or processing within the 20S core (Zmijewski et al., 2009).

#### 2.4.6. Mono- and Polyubiquitination of RPN10

The ubiquitin-receptor RPN10 can be mono- and polyubiquitinated, both having different functions. Ubiquitin-protein ligase RSP5 catalyzed the monoubiquitination of RPN10 in yeast, whereas deubiquitinating enzyme UBP2 removed this monoubiquitination (Isasa et al., 2010). Interestingly, it has been shown that RSP5 and UBP2 form a complex (Kee et al., 2006). These two enzymes controlled the monoubiquitination on three lysine residues at the N-terminus, and one lysine residue at the C-terminus of purified yeast RPN10 (Isasa et al., 2010). In human cells, also three monoubiquitination sites were identified in the N-terminus, although the lysine residues differed from yeast (Piterman et al., 2014). The C-terminal UIM-domain of RPN10, involved in the binding of polyubiquitinated substrates, was necessary for the monoubiquitination by RSP5 (Isasa et al., 2010). Monoubiquitination inhibited the capacity of RPN10 to interact with polyubiquitinated substrates, consequently inhibiting proteasome activity (Isasa et al., 2010; Piterman et al., 2014). This suggests an intramolecular interaction between the UIM-domain and RSP5/monoubiquitin.

It seems that monoubiquitination of RPN10 results in its dissociation from the 26S proteasome in yeast (Zuin et al., 2015; Keren-Kaplan et al., 2016). This is consistent with the observation that monoubiquitinated RPN10 was present mainly in a proteasome-free state in human cells (Piterman et al., 2014). The free RPN10 may bind polyubiquitinated proteins for proteasomal degradation, indicating a shuttling model regulated by cycles of ubiquitination (Keren-Kaplan et al., 2016). This may compensate the low diffusion rate of the large proteasome complex, increasing its catalytic function (Keren-Kaplan et al., 2016). This would be important under stress conditions, such as heat shock and oxidative stress, in which RPN10 was essential for the enhanced degradation of damaged and newly synthesized proteins in yeast (Medicherla and Goldberg, 2008). However, monoubiquitination of RPN10 was decreased under these stress conditions. In addition, free RPN10 binds to the extrinsic ubiquitin-receptor DSK2, which shuttles polyubiquitinated proteins to the 26S proteasome, and thereby regulates the amount of DSK2 that interacts with the proteasome (Matiuhin et al., 2008). Monoubiquitination of RPN10 by RSP5 decreased its association with the proteasome as well as DSK2, facilitating the formation of DSK2-26S proteasomes in yeast (Zuin et al., 2015). This suggests a mechanism that regulates the distribution of proteasome ubiquitin-receptors. However, it is unknown what the effect is of the DSK2-26S proteasome association.

It appears that UBE3C, a proteasome-associated ubiquitin ligase that presumably extends ubiquitin chains on substrates, extends the monoubiquitination of RPN10, since in the absence of UBE3C polyubiquitination disappeared, but mono- and diubiquitination were not affected in yeast (Crosas et al., 2006; Isasa et al., 2010). The polyubiquitination of RPN10 resulted in the degradation of the subunit (Crosas et al., 2006; Lee et al., 2014). Nevertheless, in human cells UBE3C does not seem to influence RPN10 ubqituination (Besche et al., 2014). In summary, monoubiquitination of RPN10 regulates substrate binding, and its association with the 26S proteasome and DSK2, while polyubiquitination likely results in RPN10 degradation.

#### 2.4.7. Polyubiquitination of RPN13

Ubiquitin-receptor RPN13 is also polyubiquitinated by UBE3C, although this does not regulate the degradation of the subunit. Proteasome inhibitors enhanced the association of UBE3C with the proteasome, and stimulated the polyubiquitination of RPN13 in human cells, and on purified 26S proteasomes at Lys-21 and Lys-34, which are located within or near the ubiquitin-binding PRU-domain of the subunit (Besche et al., 2014). Therefore, it is not surprising that the RPN13 modification decreased the binding of polyubiquitinated substrates, and consequently their degradation. The proteasome's ability to degrade ChT-L peptides or non-ubiquitinated unfolded substrates was not affected. Increased RPN13 polyubiquitination in cells was also observed during heat-shock, arsenite-induced oxidative stress, 19S ATPase inhibition, or RPN11 deubiquitination inhibition (Besche et al., 2014). These conditions have in common that they cause accumulation of polyubiquitinated and/or damaged proteins. Triggering ubiquitination of RPN13 by UBE3C, may prevent the binding of additional substrates to stalled proteasomes caused by protein overload, protein aggregates or damaged proteins. This might prevent damage to the proteasome, but under proteotoxic stress it may contribute to further accumulation of proteins.

The dramatic decrease in the degradation of polyubiquitinated proteins by purified 26S proteasomes upon RPN13 polyubiquitination was not expected, since substrates could still be recognized by the unaffected ubiquitin-receptor RPN10. In yeast, RPN10 and RPN13 contributed equally to the binding of ubiquitin chains (Peth et al., 2010), and in mice, RPN10 knockout was more severe than RPN13 knockout, indicating that the contribution of RPN10 is even more important than RPN13 in mammals (Hamazaki et al., 2007, 2015). Therefore, it is suggested that modified RPN13 may negatively affect RPN10. Thus, polyubiquitination of RPN13 results in decreased substrate binding, and thereby it suppresses the degradation of polyubiquitinated proteins.

### 2.5. Localization Related PMTs of the 19S Subunits

#### 2.5.1. N-myristoylation of RPT2

N-myristoylation is the irreversible linkage of myristic acid, a 14-carbon saturated fatty acid, to N-terminal glycine residues of proteins (Martin et al., 2011). This protein lipidation allows hydrophobic interactions with other proteins or membrane lipids, and plays a role in intracellular localization. N-myristoylation of the RPT2-subunit is involved in the intracellular localization of the 26S proteasome in yeast, without affecting proteasome assembly or activity (Kimura et al., 2012, 2016). In yeast cells expressing a non-myristoylated RPT2 mutant, there was a redistribution of the 26S proteasome from the nucleus to the cytoplasm (Kimura et al., 2012). The altered localization caused an accumulation of polyubiquitinated proteins in the nucleus, especially accumulation of nucleo-cytoplasmic proteins (Kimura et al., 2012, 2016). In yeast the nucleus is the major site of proteasome activity, and therefore, misfolded proteins are generally degraded in the nucleus (Prasad et al., 2010). These findings indicate that nuclear RPT2 myristoylated proteasomes play a role in the degradation of proteins in the nucleus, including misfolded proteins localized in the cytoplasm (Kimura et al., 2016). Whether nuclear degradation of cytoplasmic proteins can also be observed in mammalian cells, is not known, although it has been shown that human and mice RPT2 can be N-myristoylated (Gomes et al., 2006; Wang et al., 2007). Thus, it seems likely that the N-myristoylation of RPT2 inhibits transport of the nuclear proteasome into the cytoplasm, and therefore, is involved in the degradation of proteins in the nucleus.

#### 2.5.2. Phosphorylation of RPN8

It has been found that phosphorylation of RPN8 is involved in the localization of the subunit in breast epithelial cell lines (Thompson et al., 2004). Unmodified RPN8 was observed throughout the nucleus and cytoplasm, whereas the modified form was localized mainly in the cytoplasm. However, in malignant breast epithelial cell lines unmodified RPN8 was only present in the cytoplasm, and the modified form was not found. This suggests that there is only in the normal cell lines a mechanism that regulates nuclear localization of RPN8. Thus, RPN8 phosphorylation, and thereby localization seems to be dysregulated in cancer. Importantly, phosphorylation of RPN8 was in both cell lines induced by proteasome inhibition (Thompson et al., 2004).

Unmodified RPN8 associated with the 26S proteasome, whereas the modified form did not (Thompson et al., 2004). It has been shown that modified RPN8 can associate with (19S-unincorparated) RPN7, and likely also with other 19S-subunits (Thompson et al., 2004). Since there is a difference between the phosphorylation state of RPN8 in normal and malignant cells, the possibility raises that RPN8 regulates transcription in association with other 19S-subunits, supported by several studies that indicate that proteasome 19S-subunits have a role in gene expression, and interact with transcription regulators (Yanagi et al., 2000; Ferdous et al., 2002; Kang et al., 2002; Maganti et al., 2014). In conclusion, RPN8 can be phosphorylated, which is associated with cellular localization, and its association with the proteasome.

## 3. Modulation of the Proteasome at Transcriptional Level

Besides modifications and modulations acting directly on the proteasomal complexes, proteasome activity can also be regulated at the transcriptional level. Alterations in the expression level of proteasome subunits can modify the overall capacity and selectivity to degrade proteins dramatically. In this section important signaling pathways that affect proteasome gene expression under different conditions will be discussed: IFN-γ, NF-κB, NF-E2-related factor-2 (NRF2), and NRF1 (summarized in Table 4).

**Table 4:** Signaling pathways that effect proteasome transcription.

**Pathway**		**Trigger**		**Enhanced expression**	**Section**	**References**
IFN-γ	-JAK-STAT	Immune response		Immunoproteasome subunits^#^ PA28αβ^#^ MHC class I^#^ NOX1 and NOX4 (→ oxidative stress)	Various genes	Barton et al., 2002; Kuwano et al., 2006; Moriwaki et al., 2006; Manea et al., 2009; Zhou, 2009; Seifert et al., 2010; Grimm et al., 2012; Johnston-Carey et al., 2016; Moritz et al., 2017
	-AKT-mTOR			Various genes		
	^*^			E3 ligases and E2 ligase UBE2L6		
NRF2		Oxidative stress		20S proteasome subunits PA28αβ	3.2	Kwak et al., 2003a,b; Kapeta et al., 2010; Pickering et al., 2012
NF-κB				Immunoproteasome subunits PA28γ	3.1	Storz and Toker, 2003; Moschonas et al., 2008; Johnston-Carey et al., 2016
NRF1		Proteasome inhibition		26S proteasome subunits PA200	3.3	Meiners et al., 2003; Radhakrishnan et al., 2010; Steffen et al., 2010; Tsuchiya et al., 2013; Sha and Alfred Goldberg, 2014; Sha and Goldberg, 2016; Welk et al., 2016; Vangala and Radhakrishnan, 2019

*Important signaling pathways that effect the expression of several genes encoding proteasome subunits, or proteins involved in processes of the proteasome, under different conditions (trigger)*.

* *unknown*;

# *also oxidative stress, IFN-γ not required*.

### 3.1. Inflammatory Pathways

IFN-γ is an important activator of the immune response. It induces the expression of genes encoding proteasome subunits, or proteins associated with proteasome function. The expression of immunoproteasome subunits, PA28αβ, and MHC class I genes seems to be induced by IFN-γ via the JAK-STAT pathway (Zhou, 2009; Johnston-Carey et al., 2016; Moritz et al., 2017). Binding of IFN-γ to its receptor results in the phosphorylation of JAK1 and JAK2 (Zhou, 2009). The activated JAKs phosphorylate the receptor on specific tyrosine residues, thereby recruiting STAT1, which will lead to the dimerization and phosphorylation of STAT1. Thereafter, STAT1 will translocate into the nucleus, where it initiates the expression of IFN regulatory factor-1 (IRF-1), which induces the expression of immunoproteasome subunits, PA28αβ and MHC class I genes (Zhou, 2009; Johnston-Carey et al., 2016). Importantly, STAT1 knockout mice have lower basal expression of immunoproteasomes and PA28αβ, suggesting that basal levels are also regulated by the JAK-STAT pathway *in vivo* (Barton et al., 2002).

IFN-γ can trigger oxidative stress in cells (Watanabe et al., 2003; Seifert et al., 2010). This may be the result of increased NADPH oxidase 1 and 4 (NOX1 and NOX4) expression, via the same JAK-STAT pathway, as shown in human aortic smooth muscle cells, large intestinal epithelial cells and renal mesangial cells (Kuwano et al., 2006; Moriwaki et al., 2006; Manea et al., 2009). It seems that oxidative stress can further stimulate the JAK-STAT pathway since H_2_O_2_ also activates this pathway (Yu et al., 2006; Shimizu et al., 2008; Johnston-Carey et al., 2016). Therefore, it may enhance, in addition to IFN-γ, the expression of immunoproteasome subunits, PA28αβ and MHC class I genes (Zhou, 2009; Grimm et al., 2012; Johnston-Carey et al., 2016).

In addition, IFN-γ enhances translation of various genes via the AKT-mTOR pathway, which will increase the levels of nascent proteins, but also that of incorrectly folded proteins, known as Defective Ribosomal Products (DRiPs) (Kaur et al., 2008; Seifert et al., 2010). Damaged (newly synthesized) proteins caused by IFN-γ-induced free radicals will further enhance the pool of aggregation prone DRiPs (Seifert et al., 2010; van Deventer and Neefjes, 2010). 26S immunoproteasomes are essential for the degradation of these DRiPs which are partly polyubiquitinated, likely through the IFN-γ-induced upregulation of E3 ligases and E2 ligase UBE2L6 (Schubert et al., 2000; Seifert et al., 2010). There is evidence that the peptides generated after DRiP degradation, are ligands for MHC class I molecules (Reits et al., 2000; Schubert et al., 2000; Qian et al., 2006; Cardinaud et al., 2010; Dolan et al., 2011), although it is not clear how much DRiPs contribute to antigen presentation (Rock et al., 2014). In conclusion, IFN-γ upregulates gene expression of proteasome subunits via several pathways, thereby supporting MHC class I antigen presentation and preventing aggregate formation, which results from the increased pool of oxidized and nascent proteins (van Deventer and Neefjes, 2010).

The immunoproteasome subunits may also be upregulated via the NF-κB pathway (Johnston-Carey et al., 2016). Oxidative stress provokes the phosphorylation of protein kinase D (PKD) via the SRC-ABL pathway, which then causes the degradation of inhibitor κB (IκBα) (Storz and Toker, 2003). This results in the activation of NF-κB, which translocates into the nucleus where it induces the expression of immunoproteasome genes in cooperation with IRF-1 (Moschonas et al., 2008). This also implicates that oxidative stress can increase the expression of immunoproteasomes in the absence of an immune response, which seems consistent with the role of immunoproteasomes in the degradation of oxidized proteins (Pickering et al., 2010). In addition, it was reported that during bacterial infection, Toll-like receptor (TLR) ligands upregulate the expression of PA28γ via the NF-κB pathway in macrophages (Sun et al., 2016). In turn, PA28γ promotes NF-κB transcriptional activity by destabilizing suppressor KLF2. This positive feedforward mechanism may be important for effective defense against bacterial pathogens.

### 3.2. NRF2

The NRF2 pathway is important in the protection against oxidative stress (Taguchi et al., 2011; Tonelli et al., 2018). Under normal conditions, NRF2 is constantly polyubiquitinated via KEAP1-dependent ubiquitin conjugation, and subsequently degraded by the 26S proteasome (Kobayashi et al., 2004). Upon oxidative stress, the highly reactive cysteine residues of KEAP1 are oxidized, which causes dissociation of the KEAP1-NRF2 complex (Sekhar et al., 2010). The free NRF2 translocates into the nucleus where it heterodimerizes with small MAF proteins and binds to antioxidant/electrophile response elements (AREs or EPREs) (Taguchi et al., 2011). AREs are located in the promoter of various stress response genes, including subunits of the proteasome (Kwak et al., 2003a; Steffen et al., 2010; Pickering et al., 2012). It was shown that H_2_O_2_ treatment induces binding of NRF2 to the ARE of the β5-subunit gene, which increased the mRNA levels of this subunit (Pickering et al., 2012). It seems likely that NRF2 will also bind to the AREs of other proteasome subunits, since the enhanced expression of the 20S proteasome and PA28αβ during oxidative stress is NRF2-dependent (Pickering et al., 2012). In addition, NRF2 inducers could upregulate proteasome subunit levels and activity in mammalian fibroblasts and mouse liver tissue (Kwak et al., 2003a,b; Kapeta et al., 2010). Thus, proteasome subunit induction by the NRF2-pathway seems to increase the capacity of the cell to degrade damaged and oxidized proteins. NRF2 levels were increased in different mouse tissues in response to oxidative damage by air pollution, which may explain the accompanied elevation of 20S and immunoproteasome levels, contributing to oxidative stress adaptation Pomatto et al. (2018). However, this model was contradicted by the study of Pickering et al. (2012) which concluded that the expression of immunosubunits was not regulated by NRF2.

### 3.3. NRF1

Mild proteasome inhibition results in enhanced expression of proteasome genes, accompanied by increased proteasome protein synthesis and complex formation, which may compensate for the reduced proteasome activity (Meiners et al., 2003; Welk et al., 2016). Transcription factor NRF1 has been described as an important regulator of proteasome gene expression in response to proteasome inhibition in mammalian cells (Radhakrishnan et al., 2010). Under normal conditions, NRF1 is ER-bound and is continuously degraded via the ER-associated degradation pathway (ERAD), requiring the E3 ligase HRD1, p97 and the 26S proteasome (Steffen et al., 2010). However, when the proteasome is partially inhibited, NRF1 is proteolytically processed into a transcriptionally active form and translocates into the nucleus (Steffen et al., 2010; Sha and Alfred Goldberg, 2014). Interestingly, high concentrations of proteasome inhibitors could not induce the expression of proteasome subunits, implying completely blocked NRF1 processing by the proteasome (Sha and Alfred Goldberg, 2014). However, Sha and Goldberg (2016) showed that high concentrations of proteasome inhibitors caused NRF1 to accumulate into aggregates, thereby losing its potency to initiate transcription. Instead, it was shown that NRF1 processing does not depend on proteasomes (Sha and Goldberg, 2016; Vangala et al., 2016), but requires aspartyl proteases DDI-1/2 (Koizumi et al., 2016; Lehrbach and Ruvkun, 2016; Xiang et al., 2018). Since the enhanced transcription is dependent on ubiquitination, it was proposed that mild proteasome inhibition caused accumulation of ubiquitin moieties on NRF1. DDI2 would then bind the ubiquitin via its UBL domain and facilitate NRF1 processing (Koizumi et al., 2016). After NRF1 processing and nuclear translocation, NRF1 and the TIP60 chromatin-regulatory complex are co-recruited to the ARE-containing promoter regions of proteasome genes (Vangala and Radhakrishnan, 2019). Thereby upregulating the mRNA levels of all 26S proteasome subunits and PA200 and subsequently increasing their protein levels (Meiners et al., 2003; Radhakrishnan et al., 2010; Steffen et al., 2010; Tsuchiya et al., 2013; Sha and Alfred Goldberg, 2014; Welk et al., 2016). NRF1 also regulates the expression of its regulators p97, HRD1 and DDI creating positive and negative feedback circuits between NRF1 and these target genes (Sha and Alfred Goldberg, 2014; Xiang et al., 2018). Since NRF1 regulates the proteasome content in the cell, it is a potential therapeutic target for diseases associated with impaired or enhanced proteasome degradation by inducing or suppressing NRF1 activation, respectively (Bott et al., 2016; Weyburne et al., 2017).

NRF2 levels are also increased under proteasome inhibition, although a significant impact of NRF2 on gene-expression of proteasome genes has not been detected under this condition (Steffen et al., 2010). However, NRF2 activation upon proteasome inhibition supports the survival of cancer cells, suggesting that the NRF2 attenuates the anti-tumor efficacy of proteasome inhibitors (Lee et al., 2018; Sun et al., 2018). NRF1 and NRF2 thus differ in function, which is manifested by their different effects on proteasome gene expression. The upregulation of distinct and overlapping proteasome subunits by NRF1 and NRF2 via AREs may be explained by a difference in the binding capacity of the transcription factors to the AREs, which might also be condition-dependent (Steffen et al., 2010; Koch et al., 2011).

## Conclusion

As illustrated by the various possible modifications, proteasomes are far from being static complexes. Continuous stimuli from internal and external factors ensure a constant adaptation of proteasomes toward their cellular environment. As shown in Table 5, various conditions can lead to multiple PMTs of the proteasome. The modifications may act together to activate or inhibit the proteasome, but can also have opposite effects, illustrating the dynamic regulation of the proteasome. Since proteasome complex alterations lead to altered substrate specificities and activities to cope with specific conditions, it makes the proteasome a promising target for therapies. By forcing the proteasome pool toward specific complexes or alter activity via particular modifications, degradation capacity and specificity can be altered in particular cellular processes, thereby improving or reducing the degradation of disease-related proteins. In addition, focussing on key components involved in substrate targeting toward proteasomes would also be beneficial in a search for therapies involving proteasome degradation.

**Table 5:** Proteasome PTMs arranged according to their specific condition to which they are associated.

**Condition**	**Modifications**
Oxidative stress	PolyADP-ribosylation (20S), S-nitrosylation (20S), HNE modification (20S), phosphorylation (α4 and RPT5), S-glutathionylation (α5 and RPN2), carbonylation (RPT3), ubiquitination (RPN10 and RPN13)
Immune response	Phosphorylation (α7), 15d-PGJ_2_ modification (19S)
Proteasome inhibition	S-glutathionylation (α5 and β-subunits), phosphorylation (RPN8), ubiquitination (RPN13)
Cell cycle	Phosphorylation (α3, α4 and RPT3)
Apoptosis	Phosphorylation (α7 and RPT5)
Heat shock	S-glutathionylation (α5), ubiquitination (RPN10 and RPN13)
Osmotic stress	Phosphorylation (RPN2)
Metabolism	Methylation (20S), MGO modification (β2), O-GlcNAcylation (RPT2)
Treatment	Acetylation (several 20S subunits, and ubiquitinated α2)
-	Phosphorylation (several 20S subunits, RPT1, RPT6 and RPN6), ubiquitination (α2), N-myristoylation (RPT2)

*- Specific conditions for these PTMs are not classified*.

## Author Contributions

SK wrote the review and made the figures and tables. KG updated literature and did last editing. ER was involved in revision. SS-K supervised and revised manuscript.

### Conflict of Interest Statement

The authors declare that the research was conducted in the absence of any commercial or financial relationships that could be construed as a potential conflict of interest.
